# Partial Least Square and Parallel Factor Analysis Methods Applied for Spectrophotometric Determination of Cefixime in Pharmaceutical Formulations and Biological Fluids

**Published:** 2018

**Authors:** Ahmadreza Amraei, Ali Niazi

**Affiliations:** *Department of Chemistry, Faculty of Science Islamic Azad University, Arak Branch, Arak, Iran.*

**Keywords:** Cefixime, PARAFAC, Pharmaceutical preparations, Urine; Plasma, DATAN, Acid dissociation constant

## Abstract

In this study, the direct determination of cefixime as an anti-bacterial agent, in pharmaceutical formulations, urine and human blood plasma was conducted based on spectrophotometric measurements using parallel factor analysis (PARAFAC) and partial least squares (PLS). The calibration set was composed of fourteen solutions in the range of 0.50- 9.00 µg mL^-1^. PLS models were calculated at each pH applied to determine a set of synthetic cefixime solutions. The best model was acquired at pH 1.02 (PLS-pH 1.02). The ability of the method for the analysis of real samples was considered by determination of cefixime in pharmaceutical preparations, urine and plasma with satisfactory results. The calculated model with PARAFAC showed good prediction capability with root mean square error of prediction (RMSEP) of 0.12 for cefixime. The acid dissociation constants (p*K*_a_) of cefixime play a fundamental role in the mechanism of activity of cefixime. The p*K*_a _of cefixime were estimated by DATAN program using the corresponding absorption spectra-pH data. The calculated p*K*_a_ values of cefixime were 1.89 and 3.80 for p*K*_a1_ and p*K*_a2_ respectively.

## Introduction

**Figure 1:**
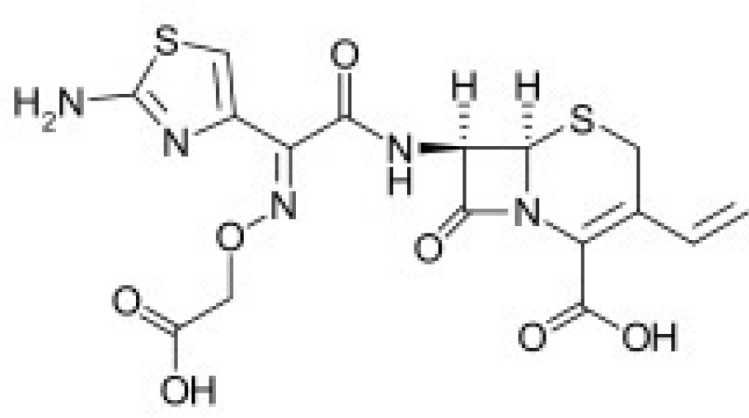
Chemical structure of cefixime

Cefixime is a cephalosporin related to the β-lactam class of antibiotics, used orally to treat infections due to susceptible Gram positive and Gram negative bacteria (1, 2) Figure 1. Cefixime was applied against susceptible bacteria causing infection of the middle ear, tonsillitis, throat infections, laryngitis, bronchitis, urinary tract infections, gonorrhea, and pneumonia (3).

The p*K*_a_ plays an important role in the mechanism of activity of various biological fluids, primarily the blood, and its capability to interact with components of these fluids and other drugs can be investigated (2). The mean half-life of cefixime in human plasma is about 3-4 h. The plasma levels of cefixime are proportional to the dose given. For this reason, a special and sensitive analytical method is necessary especially in case of biological fluids investigations, because of the existence of low drug concentrations in this matrix, unless a hydrolysis stage is attained (4). Several procedures have been reported for the determination of cefixime in various matrices applying high-performance liquid chromatography (5), liquid chromatography–tandem mass spectrometry (6), voltammetry (7), fourier transform infrared spectroscopy (8). Spectrophotometric methods are the most commonly used methods due to common availability of instrumentation, wide application range, experimental speed, precision and accuracy of the technique (9-11). However, at present, few methods have been proposed for the determination of cefixime using spectrophotometry. Therefore, with the growth in the manufacturing and consumption of drugs that contain cefixime, it becomes interesting to develop sensitive analytical method for its determination (12). In recent decades, two-and three-way analysis was proposed in the scope of analytical chemistry (13, 14). Theory and use of PARAFAC (15, 16) and PLS (17) in spectrophotometry has been communicated by several researchers (18-20). In addition, several determination based on the use of these methods to spectrophotometric data have been offered (21-24). In this study, a method for quantitation of cefixime in pharmaceutical formulations, urine and plasma based on direct UV spectrophotometric measurements have been reported. The study in the pH range from 1.0 to 12.0 and with a linear concentration range from 0.50 to 9 µg mL^−1 ^was performed. The PLS at several pH and PARAFAC were used for UV spectral deconvolution and cefixime determination. During the spectra deconvolution step by PARAFAC, core consistency diagnostic (CORCONDIA) procedure was used to determine the number of different species present in the data set. In PARAFAC quantitation, the sample factor loadings were used to establish a linear relationship with cefixime concentrations and good results were obtained for samples at low µg mL^−1^ concentrations. The acid dissociation constants (p*K*_a _values) can play a fundamental role for perception and determining chemical phenomena such as reaction rates, biological activity, biological uptake, biological transport, and environmental fate (25). Thus, we are interested in acquiring acidity constants of cefixime and also using their results in pH selection for PARAFAC data utilization. The acid dissociation constants of cefixime at 25 °C and ionic strength of 0.1 mol­ L^-1^ have been measured spectrophotometrically. DATaANalysis (DATAN) software was used for estimation of acid dissociation constants. Outputs of DATAN software were p*K*_a_ values, number of principal components, concentration distribution diagrams, and net spectrum of each assumed species. The theory and usage of the physical constraints method was investigated by Kubista *et al.* in different papers (26-28). To verify the predictive ability of these models, the RMSEP and RSEP were used.

$\mathrm{RMSEP}=\sqrt[{2}]{\frac{\sum_{1}^{n}{\left(y_{\mathrm{pred}}-y_{\mathrm{obs}}\right)}^{2}}{n}}$

(1)

$\mathrm{RSEP}(\%)=100\times \sqrt[{2}]{\frac{\sum_{i=1}^{n}{\left(y_{\mathrm{pred}}-y_{\mathrm{obs}}\right)}^{2}}{\sum {\left(y_{\mathrm{obs}}\right)}^{2}}}$

(2)

Where y_pred _is the predicted concentration, y_obs_ is the observed value of the sample, and n is the number of samples in the validation set.

## Experimental

*Reagents*

All the reagents were of analytical reagent grade. Cefixime, phosphoric acid, acetic acid, boric acid, hydrochloric acid, potassium nitrate and sodium hydroxide were purchased from Merck. Stock standard solution of cefixime, 1000 µg mL^−1^ was prepared by dissolving the cefixime in methanol. All the solutions were prepared in deionized water. Universal buffer solutions in pH range from 1.0 to 12.0 were prepared (29).

*Instrumentation and software*

A Perkin Elmer (Lambda 25) spectrophotometer equipped with a 1-cm path length quartz cell was employed for UV spectra acquisition. Spectra were acquired between 2.0 and 350 nm (1 nm resolution). A Metrohm 692 pH-meter furnished with a combined glass-saturated calomel electrode was calibrated with at least two buffer solutions at pH 3.00 and 9.00. All evaluations for this work were carried out in MATLAB (Version 7.8.0 (R2009), MathWorks Inc.) run on a Sony personal computer. The *N*-way toolbox for MATLAB version 2.1, available at http://www.models.kvl.dk/source, was employed for PARAFAC calculations, while PLS calculus was carried out by the PLS-Toolbox, version 2.0 (Eigenvector Technologies).

*Procedure*

Appropriate amounts of standard solutions were added in a 10 mL volumetric flask and diluted to the final volume with deionized water and universal buffer in pH range from 1.0 to 12.0. The final concentration of these solutions varied between 0.50 to 9.00 µg mL^−1^ for cefixime. 

*Tablets formulation preparation*

The pharmaceutical preparations considered had the following composition per tablets: Exir (Iran), 200 and 400 mg of LOPRAX and Dana (Iran), 200 and 400 mg of Zaxime. Five tablets of each pharmaceutical formulation were weighed individually to an average weight. The tablets were finely powdered and mixed, and a mass corresponding to one tablet for each formulation was weighed and dissolved in 100 mL of methanol-water (10:90, v/v) in a volumetric flask. An aliquot of each sample was added into a cuvette containing 2.50 mL of the respective buffer with the specified pH (4). The spectra were acquired under the same conditions in previously described procedure. All these analysis were carried out in triplicate.

*Analysis of urine samples*

Urine spiked with cefixime was prepared using the following method; the samples were centrifuged for 10 min at 3800 rpm and filtered through a cellulose acetate filter (30). Resultant residue was dissolved in universal buffer (at different pH) into a 10 mL volumetric flask and diluted to the mark with buffer solution.

*Analysis of plasma samples*

Blood plasma spiked with cefixime was treated by diluting aliquots of the stock standard cefixime solution with the human plasma. The plasma sample was prepared by direct protein precipitation with acetonitrile (31) and then it was added into 10 mL volumetric flask and diluted to mark by universal buffer (at different pH).

## Results and Discussion

*Determination of acid dissociation constants*

**Figure 2:**
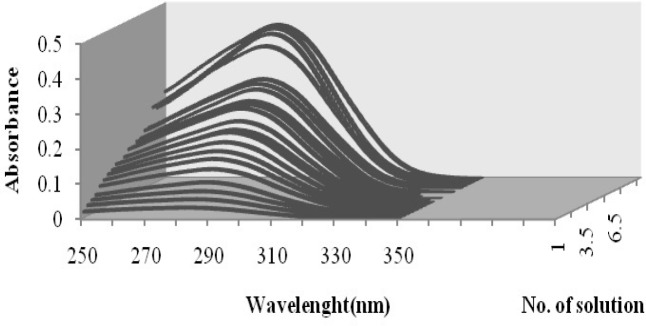
Absorption spectra of cefixime at different pH values: (1) 1.00, (2) 1.87, (3) 2.00, (4) 2.15, (5) 2.25, (6) 2.36, (7) 2.50, (8) 2.70, (9) 2.90, (10) 3.02, (11) 3.12, (12) 3.23, (13) 3.32, (14) 3.65, (15) 3.86, (16) 4.15, (17) 5.31, (18) 6.00, (19) 6.50, (20) 7.03, (21) 7.54, (22) 8.03, (23) 8.76, (24) 9.23, (25) 9.90, (26) 10.70, (27) 11.25

The absorption spectra of cefixime in water at different pH values at 200–350 nm intervals were digitized. Sample spectra of cefixime at different pH values in water are presented in Figure 2.

**Figure 3:**
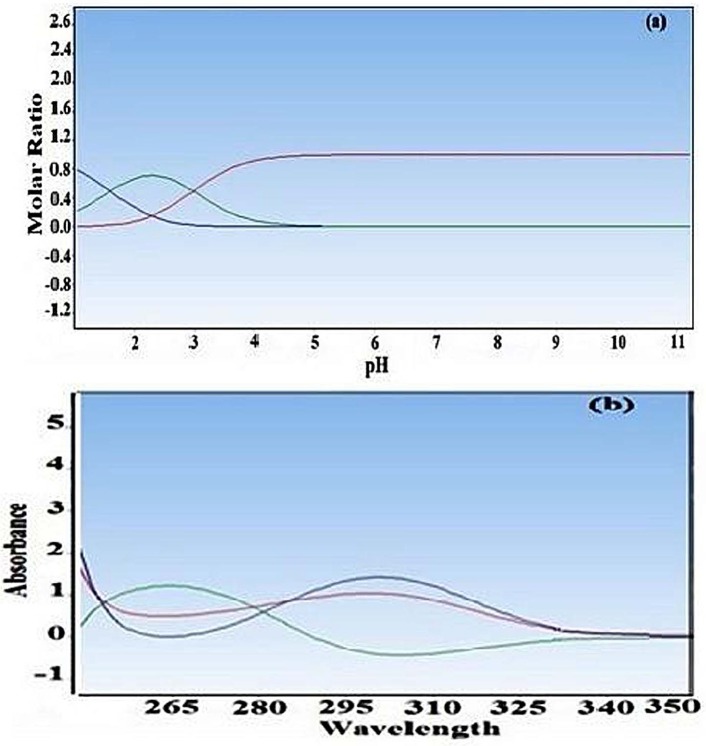
(a) Distribution of major species of crfixime as function of pH for the spectral data of Figure 2. (b) The pure absorption spectra of different form of cefixime

The principal component analysis of all absorption data sets acquired at several pH clearly shows at least three significant factors. The p*K*_a_ values of cefixime were studied spectrophotometrically at 25 °C and ionic strength of 0.1 M. The p*K*_a_ of cefixime was estimated by DATAN software using the corresponding absorption spectra-pH data. The outputs of DATAN software are p*K*_a_ values, number of significant factors, concentration distribution diagrams and net spectrum of each assumed species. The p*K*_a_ values of cefixime were previously reported as p*K*_a1_ = 2.1 and 

p*K*_a2_ = 3.73 (2). The second p*K*_a_ corresponds to the protonation of the nitrogen in the amine group. However, both N-H group and O-H group were offered to be possible deprotonation sites. The concentration distribution diagram and pure spectra are shown in Figure 3.

*PARAFAC analysis*

**Figure 4:**
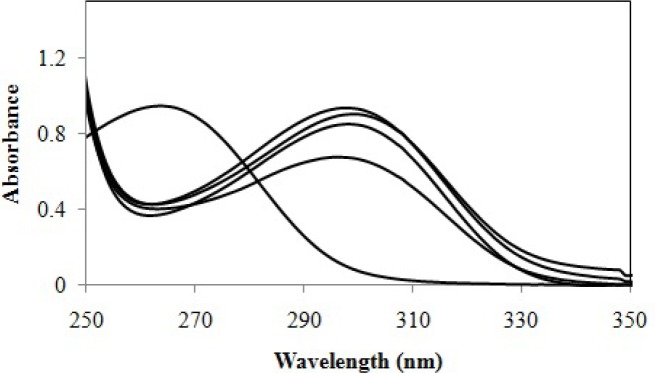
Spectral data used in determination of cefixime by PARAFAC, at pH 1.02, 2.36, 3.34, 4.05 and 7.20

The major benefit of three-way multivariate calibration is that it permits concentration information of an original component to be obtained in the presence of any number of unmodeled interfering components. Thus, it is very useful for the elimination of many analytical problems involving a complex matrix. The data (Figure 4) have been arranged in a three-way array 21 × 150 × 5, composed of 21 solutions, with different cefixime concentrations (Table 1), in the rows, 150 wavelengths in the columns and 5 pH values in the slices.

**Table 1:** Concentration data of the calibration and prediction set of cefixime for PARAFAC and PLS models (µg mL^−1^)

**Calibration**	**Concentration**	**Calibration**	**Concentration**	**Prediction**	**Concentration**
C1	0.50	C8	5.50	P1	0.90
C2	1.25	C9	6.00	P2	2.25
C3	1.75	C10	6.50	P3	3.00
C4	2.75	C11	7.25	P4	4.00
C5	3.50	C12	7.75	P5	5.25
C6	4.25	C13	8.50	P6	7.00
C7	4.75	C14	9.00	P7	8.00

No preprocessing (mean centering or autoscaling) was applied to the data. By applying PARAFAC, a primary definition of the number of factors to make the model is necessary. This selection is of basic importance because all results on the deconvolution and quantitation will be dependent on this number of factors (Table 2).

**Table 2:** Fit values and core consistency diagnostic values in percentages vs. the number of components in the PARAFAC model

**Number of factors**	**1**	**2**	**3**
Fit (%)	100	99.99493	99.99577
CORCONDIA (%)	92.96639	93.29524	86.79659

**Figure 5:**
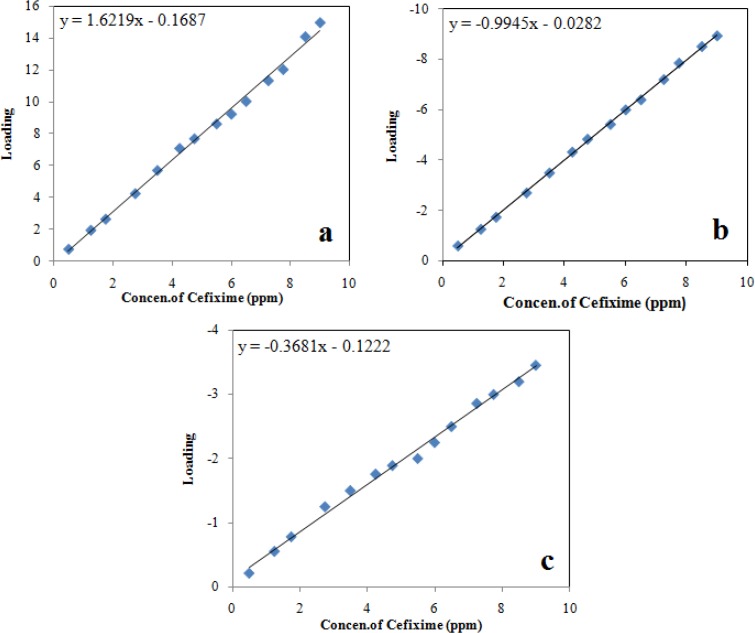
Calibration graphs for cefixime: (a) first loading of C-loadings, (b) second loading of C-loading, and (c) third loading of C-loading

In PARAFAC, it is possible to utilize various constraints such as non-negativity, unimodality or orthogonality. In this study, an unconstrained model preferred as more realistic results can be acquired. Unconstrained PARAFAC models of the cefixime data at various pH were expanded using one to five components and the percentage of fit was applied as the primary approach to choose the number of factors. The decomposition of the three-way data by PARAFAC gives rise to three loading matrices, one of which, C, corresponds to the sample mode. The C-loading are the relative concentrations of the cefixime in the solutions. In the calibration step, these loadings are plotted against the real concentrations of cefixime to a linear calibration. By plotting these loadings (C-loading) versus real concentrations of cefixime, three calibration curves obtained that are shown in Figure 5. Linear regression results and standard deviation of results, line equations and correlation coefficient are presented in Table 3.

**Table 3:** Statistical parameters of the linear relationship between the proportion loadings calculated by PARAFAC and the true concentration of cefixime

	**First loading of C-loading (first calibration)**	**Second loading of C-loading (second calibration)**	**Third loading of C-loading (third calibration)**
Number of data points	14	14	14
Intercept	-0.1687	-0.0282	-0.1222
Standard deviation of intercept	2.65	0.012	3.27
Slope	1.622	-0.9945	-0.3681
Standard deviation of slope	0.473	0.011	0.487
Correlation coefficient*	0.9945	0.9994	0.9945
Standard deviation of regression	0.312	0.063	0.100

* *P *< 0.001 at 95% confidence level.

In the prediction step, this regression line can then be applied to predict the concentration of cefixime in latter test samples. In PLS method, according to an experimental design (Table 1) Fourteen solutions were used to create the models (calibration set) and another seven solutions to validate them (validation set). The results obtained by applying PARAFAC and PLS to seven synthetic samples are presented in Tables 4 and 5. Tables 4 and 5 also show the RMSEP and RSEP. The prediction results for cefixime are very good recovery. 

**Table 4:** Added value and found results of the prediction set of cefixime using PARAFAC method (µg mL−1)

**Added cefixime**	** First calibration**		** Second calibration**		** Third calibration**	
	**Found**	**Recovery (%)**	**Found**	**Recovery (%)**	**Found**	**Recovery (%)**
0.90	1.45	161.11	0.93	103.33	0.40	44.44
2.25	3.35	149.99	2.19	97.33	1.00	44.44
3.00	4.95	165.00	3.10	103.33	1.50	50.00
4.00	6.50	162.50	4.10	102.50	1.74	43.50
5.25	8.18	155.81	5.17	98.48	2.03	38.66
7.00	11.00	157.14	6.77	96.71	1.71	24.43
8.00	12.82	160.25	8.13	101.62	3.50	43.75
RMSEP	2.90		0.12		3.11	
RSEP	59.2		2.41		63.26	

This value shows considerable superiority with respect to PLS method at different pH (Table 5).

**Table 5:** Added value and found results of the prediction set of cefixime using PLS method at different pH (µg mL−1)

**Added**	**PLS-pH 1.02**		**PLS-pH 2.36**		**PLS-pH 3.34**		**PLS-pH 4.05**		**PLS-pH 7.20**	
	**Found**	**Recovery**	**Found**	**Recovery**	**Found**	**Recovery**	**Found**	**Recovery**	**Found**	**Recovery**
0.90	0.87	96.66	0.89	99.00	0.66	73.33	0.88	97.77	0.90	100
2.25	1.96	87.11	2.34	104.00	1.40	62.22	1.90	84.44	2.34	104.00
3.00	3.30	110	3.30	110.00	2.20	73.33	3.73	124.33	3.07	102.33
4.00	3.87	96.75	4.14	103.50	3.67	91.75	3.94	98.50	4.27	106.75
5.25	5.30	100.95	5.02	95.62	4.64	88.40	5.03	95.81	5.16	98.30
7.00	7.10	101.43	6.99	99.85	6.86	98.00	6.70	95.71	7.03	100.43
8.00	8.01	100.12	8.20	102.5	7.91	98.87	7.25	90.62	8.35	104.35
No. of factor	2		2		2		3		3	
RMSEP	0.133		0.178		0.525		0.375		0.176	
RSEP	2.70		3.60		10.62		7.60		3.56	

*PLS analysis*

*Calibration and validation*

The multivariate regression is a useful tool for determinations, because it extracts more valuable information from the data set and allows the making of more satisfactory models. Thus, it was decided to carry out a multivariate regression using PLS models created for each pH value individually and compare with PARAFAC model. To have a satisfactory model (Table 1) 14 solutions were used to create the models (calibration set) and another seven solutions to validate them (validation set). The models were validated using cross validation. The RMSEP and RSEP values were applied as parameters for comparing the models.

*Selection of the optimum number of factors*

To help determine significant number of factors in the PLS calibration model, cross validation methods were applied. The predicted concentration of the calibration samples were calculated and compared with the actual concentrations, and the predictive residual error sum of squares (PRESS) was computed. A graph of PRESS versus number of factors for each sample determines a minimum value for optimum number of factors.

*Determination of cefixime in synthetic mixture*

The predictive capability of both two- and three-way models at each pH was determined using seven synthetic mixtures (their compositions are presented in Table 5). The results acquired by applying PLS at each pH to six synthetic samples are presented in Table 5. Table 5 shows the RMSE and RSEP. It can be observed that PLS model at pH 1.02 is the best model.

*Determination of cefixime in pharmaceutical formulations and biological fluids*

In order to display the analytical performance of the suggested methods, three calibration graphs acquired from PARAFAC and PLS model at pH 1.02 were used to determine cefixime in real samples (pharmaceutical formulations) and complex matrices, *i.e.* urine and human plasma. The evaluation shows that satisfactory recovery for cefixime could be obtained (Tables 6 and 7) by applying the proposed procedures. Recoveries of the determination of cefixime are summarized in Tables 6 and 7. The mean recoveries in pharmaceutical formulations (Exir and Daana Tablets) and urine and human plasma are presented in Tables 6 and 7, respectively.

**Table 6:** Determination of cefixime in pharmaceutical preparations using the PARAFAC and PLS-pH 1.02 models

**Pharmaceutical preparations**	**Added value (µg mL** ^-1^ **)**	**Amount found (PARAFAC)**	**RSD (%)**	**Recovery (%)**	**Amount found (PLS-pH 1.02)**	**RSD (%)**	**Recovery (%)**
Exir^a^	4	3.93 ± 0.106	2.50	98.25	4.17 ± 0.18	4.51	104.25
	2	2.20 ± 0.091	4.02	110.00	2.37 ± 0.18	4.51	118.50
Dana^b^	4	3.88 ± 0.140	3.50	97.00	4.32 ± 0.20	4.48	101.41
	2	2.10 ± 0.115	5.75	109.00	2.23 ± 0.410	5.20	111.50

a Tablet (from Exir Ltd., Iran ).

b Tablet (from Dana Ltd., Iran ).

**Table 7:** Determination of cefixime in urine and human plasma using PARAFAC and PLS-pH 1.02 models

**Type of samples**	**Added value ** **(µg mL** ^−^^1^ **)**	**Amount found (PARAFAC)**	**Recovery (%)**	**RSD (%)**	**Amount found (PLS-pH 1.02)**	**Recovery (%)**	**RSD (%)**
Plasma sample 1	5.00	4.84	97.00	2.31	4.20	84.00	3.10
Plasma sample 2	3.50	3.50	100	3.02	3.15	90.00	4.45
Urine sample 1	2.00	2.10	100.50	5.13	1.73	86.50	5.30
Urine sample 2	6.00	5.78	96.33	2.15	5.32	88.66	4.78

## Conclusion

In this study, the determination of cefixime in biological fluids and pharmaceutical formulations was conducted based on ultraviolet spectrophotometry detection applying PARAFAC and PLS regression. The study was carried out in the pH range from 1.0 to 12.0 and with a linear concentration range from 0.50 to 9.00 µg mL^−1^ of cefixime. Multivariate calibration models applying PLS at various pH and PARAFAC were discussed for ultraviolet spectral deconvolution and cefixime determinant. The best results for the system were obtained with PARAFAC and PLS at pH 1.02. The ability of the method proposed for the analysis of actual samples was considered by determination of cefixime in pharmaceutical formulations, urine and plasma with satisfactory recoveries. Moreover, the acid dissociation constants of cefixime at 25 °C and ionic strength of 0.1 M have been determined spectrophotometrically. Eventually, it can be concluded that the model proposed by the PARAFAC method has more prediction capability especially for real samples in comparison to PLS method, which clearly shows that the tolerance limit of three-way calibration methods for matrix effect is better than that of the two-way methods.
